# Evidence on Milk Consumption and Production Linkages from Rural Bihar, India

**DOI:** 10.1016/j.cdnut.2024.102122

**Published:** 2024-02-24

**Authors:** Rekha Ravindran, Sumathi Swaminathan, Patrick Webb, Anura V Kurpad, Tinku Thomas

**Affiliations:** 1 Division of Epidemiology and Biostatistics, St. John’s Research Institute, Bangalore, India; 2 Department of Liberal Arts, Indian Institute of Technology Bhilai, Bhilai, India; 3 Division of Nutrition, St.John’s Research Institute, Bangalore, India; 4 Friedman School of Nutrition Science and Policy, Tufts University, Boston, MA, United States; 5 Department of Physiology, St. John’s Medical College, Bangalore, India; 6 Department of Biostatistics, St.John’s Medical College, Bangalore, India

**Keywords:** milk, consumption, production, small farm households, rural India

## Abstract

**Background:**

Milk is an important source of protein for many Indian households. However, milk intake is very low. Hence, it is necessary to examine production–consumption linkages of milk within the paradigm of accessibility, availability, and affordability.

**Objectives:**

This study examined linkages between milk consumption and production, accounting for sales and factors associated with production investments in rural Bihar, a major milk-producing state of India with very poor nutritional status.

**Methods:**

A panel of households from the Gaya and Nalanda districts of Bihar were surveyed: the first round in July and August 2019 (*n* = 2026 households) and the second round from December 2019 to January 2020 (*n* = 2001 households). Data were collected on household consumption, production, and sale of milk, as well as other foods. The study examines the consumption–production linkage of milk and the association of dietary diversity with consumption from own production, with households as the unit of analysis. Ordinary least square regression analysis of average monthly household milk consumption was used to identify factors associated with milk consumption, particularly milk production.

**Results:**

The median (Quartile 1, Quartile 3) per capita milk consumption per day was 83.3 (41.6, 166.6) mL in the milk-consuming households. Average monthly household milk consumption in liters was higher in milk-producing households [β: 7.1; 95% confidence interval (CI): 6.1, 8.1] than households relying on market purchases. Household milk consumption was higher in the third tertile of milk production than the first tertile of production (β: 14.3 L/wk; 95% CI: 12.1, 17.2) and lower in the highest tertile of household sale quantity (β: −8.8 L/wk in tertile 3, 95% CI: −12.7, −5) than the first tertile of household sale quantity of milk.

**Conclusions:**

The study provides evidence that consumption of milk in rural households is associated with own production such that households with higher production consume more. However, sale preferences restrict the quantity of milk consumed in milk-producing households.

## Introduction

Among micronutrient-rich foods, dairy products are consumed at relatively low levels in low-income countries [1]. Dairy products are significant sources of animal-sourced proteins, essential amino acids, and other micronutrients, including zinc, vitamin A, and calcium. Due to its rich content of highly digestible proteins, minerals, and insulin-like growth factor-I [2], milk has been identified as a significant food component for stimulating linear growth in children [3]. However, using the Global Dietary Database, Miller et al. [4] reported that the mean milk consumption per person in India was ∼100 g/d, which is far below 1 serving of 250 g/d. The highest mean intakes per person were reported in Mexico, the United Kingdom, the United States, and France (188–206 g/d), whereas the lowest were in Nigeria, China, Bangladesh, and DR Congo (31–37 g/d).

In rural areas, household production is reported to be a key driver of milk consumption [[5], [6], [7]]. Evidence suggests a positive association between milk production and consumption [1,8]. Not only does milk production enhance the availability of milk for own consumption, but the sale of milk can supplement household income [3] and support the purchase of additional food from the market [5]. Household production for own consumption is therefore regarded as a critical factor to ensure household dietary diversity [7,9]. Nevertheless, the consumption–production association is complex, implying that merely improving production by farmers does not necessarily improve their own consumption, mainly because it is also an income generator [5]. Hence, it is significant to examine how sale preferences affect the consumption of milk in rural households.

Across Indian states, Bihar has the highest share of the country’s population (51.9%) who are classified as “multidimensionally poor” by the Multidimensional Poverty Index [10]. This state is predominantly agrarian, with animal husbandry contributing significantly to rural incomes. According to National Dairy Development Board, Bihar accounted for 5% of national milk production (ranking ninth in milk production in the country) in 2017–2018 [11]. Parappurathu et al. [6] documents that 42% to 86% of milk consumption in the state is from household/own production.

This study aimed to understand factors associated with milk consumption in Bihar, particularly household production of milk and market purchase of milk. The effect of milk consumption from own production on household dietary diversity was also explored. The study investigated the association between the quantity of milk consumed and the quantity of milk produced and sold in milk-producing households. Factors associated with quantity of milk production were also examined in a subset of milk-producing households.

## Methods

### Data for the study

A panel of households with children from the Gaya and Nalanda districts of Bihar was surveyed in 2 rounds: first, in the period from July to August 2019 (*n* = 2026 households) and second, a follow-up survey from December 2019 to January 2020 (*n* = 2001 households), henceforth referred to as Round 1 and Round 2, respectively. These months were chosen to represent the seasonal variation in production of foods, including milk production, which is known to be higher in winter (Round 2) than in rainy and summer seasons, which were captured in Round 1 [12]. These districts were chosen because considerable production of milk was reported in these districts. In the year 2017–2018, Gaya and Nalanda reported bovine milk production of 145,000 MT and 148,000 MT, respectively [11].

**FIGURE 1:**
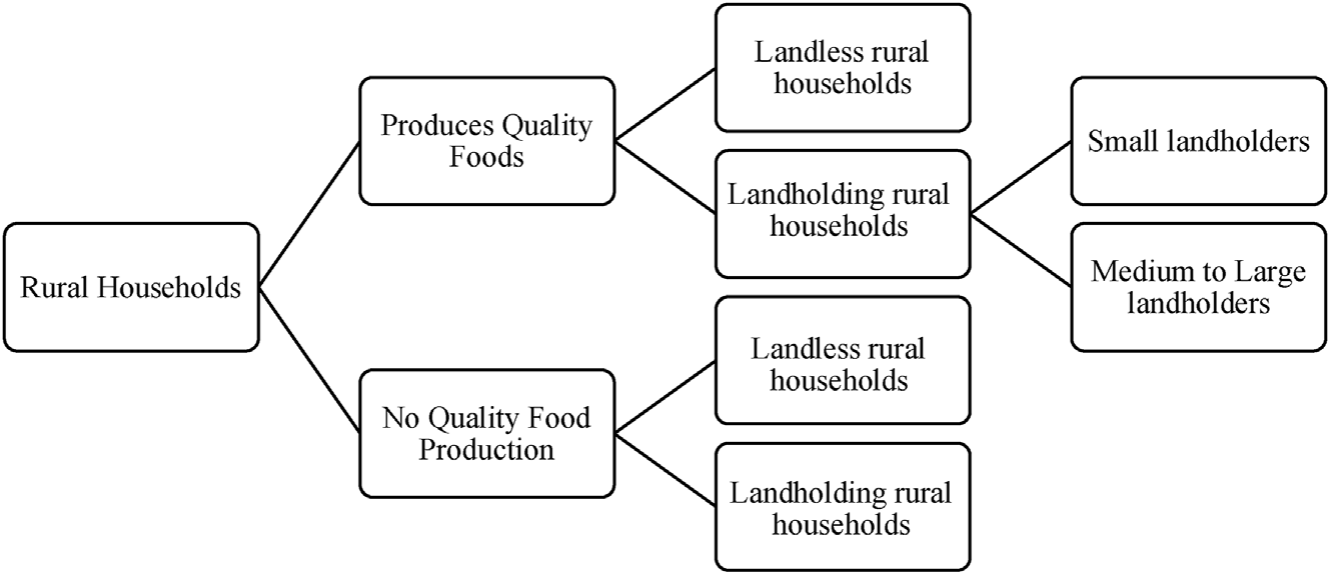
Diagrammatic representation of sampling framework for Round 1 survey.

Rural households containing ≥1 child aged 6 to 59 mo were sampled. Households were selected by a multistage cluster sampling method where each administrative village served as a cluster with census as the sampling frame for randomly selecting 101 villages from each district. Approximately an equal number of villages was selected from 0 to 5 km, 6 to 15 km, 16 to 30 km, and >30 km distance bands from the district headquarters to allow for varying distances to a large market at the district headquarters. About 10 households per village were sampled using the Random Walk method. The village was divided into 5 segments from the center of village based on the village map such that a segment was in the shape of a pie. Within each segment, 4 landmarks were designated, and 1 landmark was randomly chosen. A bottle was centrifuged at that point, and the sampling was initiated in the direction pointed to by the mouth of the bottle. This exercise was followed in each segment. Within each segment the first 2 consenting households with a child <5 y was sampled. We sampled both landless households and landholding households that were or were not engaged in producing nutrient-dense foods such as pulses, eggs, chicken, milk, and green leafy vegetables (Figure 1). The households considered in the “landless but produce nutrient-dense foods” category were landless households that produced eggs, milk, chicken, and green leafy vegetables (which do not need much land) as well as those landless households that cultivate on other people’s farms. Landholders involved in quality food production were further stratified as small land holders (with land ≤3 acres) and medium to large land holders (with land >3 acres).

Household data on the consumption of food and nonfood goods and services were collected using a household consumption questionnaire used in the national consumer expenditure survey [13]. Round 1 data were used to examine patterns and factors associated with milk consumption from different sources, such as consumption from own production and/or from market purchase. In Round 1, 614 households consumed milk from their own production, and 1005 households relied on market purchases for milk consumption. In-depth study of milk production was conducted in a random sample of 350 milk-producing households in the Round 2 survey. Among these 350 producers, 112 households sold milk, and details of milk sales were collected from them. The Round 2 sample, from which the details of milk production were collected (350 households), was a representative subsample of the entire sample of milk producers in Round 1 (614 households), as seen in Supplemental Table 1.

The quantity of milk, primarily liquid milk, consumed in the previous week by the 2026 households as recorded in the Round 1 survey was used in this analysis. The quantity of consumption of other milk products (such as curd, buttermilk, ghee, butter, and cottage cheese) was excluded due to relatively low consumption (only 31% of households reported any consumption).

A pilot study on 30 households in each district was conducted to examine the feasibility of the production survey and to validate the questionnaires. The household characteristics related to production of milk had very little variability, so a sample size of 300 households was deemed sufficient for the production survey. The details of milk produced in the last 30 d by milk-producing households were captured in the production survey during Round 2 in ∼50% of producing households (350 sampled of 1208 producing households). This subsample was selected randomly.

#### Household diet diversity

Dietary diversity is defined as the variety of foods across and within food groups consumed over a given reference period to ensure the required intake of essential nutrients for being in a state of good health [6]. The Household Dietary Diversity Score (HDDS) is constructed based on the number of food groups consumed by the household during the previous 7 d. Food items reported as consumed were classified into 12 different food groups as proposed by the FAO [14]. The 12 food groups are cereals; roots and tubers; legumes, nuts, and seeds; vegetables; meat; eggs; fish; fruits; milk and milk products; oils and fats; sweets; and spices, condiments, and beverages. Each food group added 1 score point toward HDDS if any food item from that group was consumed by any member of the household in the given 7-d period.

### Statistical methods

#### Milk consumption: availability and accessibility

The role of household production of milk compared with market purchase on household consumption of milk was examined in Round 1 data (*n* = 1946 households with complete data on all covariates) using ordinary least square (OLS) model to examine how availability was associated with milk consumption.

(1) $$Y_{i}=\beta_{1\;}\;Source\;of\;Milk\;Consumption+\underline{\gamma }\underline{X}+\in_{i}$$

In the above model, the dependent variable *Y* represents the quantity of weekly milk consumption by the household (represented by i in the model). When the household consumed from own production, it was considered as more available. Hence, the primary factor of interest was the source of milk consumed, classified as market purchase, own production, consumption from both these sources, and nonconsumers. In the above model, the consumption from market purchase was considered as the reference category. Potential confounders considered were household income (as quintiles of monthly per capita consumer expenditure [MPCE]); number of children in the household; number of adults in the household; weekly quantity consumption of food items such as rice, wheat, vegetables, eggs, fish and meat; household is or is not a beneficiary of the public distribution system (PDS); agricultural land owned; educational attainment of household head; and sex of household head, represented by the vector *X* in Equation *1*. Access to cold storage (such as own refrigerator) was not considered a potential confounder in the analysis as only 6.37% households in rural Nalanda and 5.53% households in rural Gaya have access to a refrigerator as per the National Family Health Survey (NFHS-5, 2019–21) report [15]. In the above model, $\in_{i}$ represents the error term. In this model, we also account for the effect of clustering at the village level by considering cluster robust variance estimates.

Next, to understand the role of accessibility in consumption of milk among households that purchased milk for consumption (*n* = 951), we considered the following OLS regression model:

(2) $$Y_{i}=\beta_{0}+\beta_{1\;}purchase\;inside\;village+\beta_{2\;}distance\;to\;market+\underline{\gamma }\underline{X}$$

In the above model, i represents the household. The dependent variable *Y* indicates the quantity of weekly milk consumption by the household. Accessibility was assessed by 2 variables, namely, distance to market and the binary variable “purchase inside village,” which indicated whether or not the point of purchase is inside the village. Data were not available in the survey about distance to potential market for households that did not consume milk or those that did not purchase milk. Hence, only those households that purchased milk for consumption (*n* = 1005) were considered in this analysis. In Equation *2*, we considered the same set of confounders as in Equation *1*, which is captured through vector $\underline{X}$ in Equation *2*. Village level clustering effect is accounted in this model by accounting for cluster robust variance estimates.

Thus, Equations *1* and *2* combinedly examined the role of availability and accessibility of milk on household milk consumption. The data for these analyses were from the Round 1 consumption survey.

#### Association between milk consumption from own production and household diet diversity

Household production for own consumption is often perceived as the direct route to improved household food security and nutritional status [16]. We used the Round 1 survey to analyze the effect of milk consumption from own production on households’ quality of diet, measured by household dietary diversity. Probit regression analysis of the binary variable good diet diversity (*DD*, 1=Good, 0=Poor) was performed to examine the association of household milk consumption from own production on household dietary diversity. Good household diet diversity was identified as HDDS ≥10 (which is the median of HDDS score and assigned the value 1), and poor was assigned the value 0 if HDDS <10.

(3) $${DD}_{i}=\beta_{0}+\beta_{1\;}\;milk\;consumption\;from\;own\;production+\underline{\gamma }\underline{X}$$

Here, i represents households with milk consumption from own production, coded as 1 if the milk was derived from own production and 0 otherwise. In the above framework, the confounding effects of household-specific characteristics (wealth quintile, number of children and adults, PDS beneficiary, agriculture land, education of household head, sex of household head) were accounted for in the model (represented by vector *X* in Equation *3*).

#### Association between quantity of milk consumed and produced

We pursued our empirical analysis by examining the association of quantity of milk consumed with reported quantity produced and sold among 350 milk-producing households from Round 2 consumption and production data. We did not use Round 1 consumption data for this analysis as there could be seasonal differences in consumption and production and the production in Round 2 period could be associated with consumption during the same period. In this model as well, we considered household weekly milk consumption quantity as the dependent variable. The milk producers were classified into tertiles based on quantity of milk produced (represented by categorical variable “production tertile” in Equation *4*), with the lowest tertile as the reference category. We also examined how consumption is affected if the households sold milk or not, which was coded as 1 if the household sold milk and 0 otherwise. The relationship between quantity of household milk consumption and quantity of milk production was examined using the following OLS model (Equation *4*), and we did not account for the longitudinal nature of the study as the production and consumption data used for this analysis was exclusively from this Round of data collection.

(4) $$Y_{i}=\beta_{0}+\beta_{1\;}{production\;tertile}_{i}+\beta_{2\;}{Milk\;sold}_{i}+\underline{\gamma }\underline{X}+\mu_{i}$$

Here, *Y* is the household weekly milk consumption quantity, and i represents the household. The coefficients for production and sales are accounted for by household income (MPCE), number of children, number of adults, PDS beneficiary, agriculture land, education of household head, sex of household head (vector *X* in the model). Production of cereals and pulses were also confounders in this analysis and was considered in the model along with other confounders represented by vector X in Equation *4*.

In addition, a separate model (Equation *5*) was considered to examine the association of quantity of milk sold on milk consumption in milk-producing households. Here, categorical variable sale tertile (which indicates the classification of households based on quantity of milk sold) was considered, with tertile 1 of quantity of milk sold as the reference category; other categories are tertile 2, tertile 3, and nonsellers. The same set of confounder variables (vector *X*), as in Equation *4*, were considered in Equation *5*.

(5) $$Y_{i}=\beta_{0}+\beta_{1\;}p{rouction\;tertile}_{i}+\beta_{2\;}{sale\;tertile}_{i}+\underline{\gamma }\underline{X}+\mu_{i}$$

#### Factors associated with milk production

We used Round 2 survey production data to ascertain the input–output relationship in milk production. The Cobb–Douglas form of production function gives the following specification in input–output relationship.

(6) $$Y=\alpha \;\prod_{i=1}^{k}Z_{i}^{\beta_{i}}\;e^{u}$$

In Equation *6*, *Y* represents the output (quantity of milk produced), and $Z_{i}$ denotes the input variables. The linear transformation of the above production function is as follows:

(7) $$\ln\;Y=\ln\;\alpha +\beta_{i}\sum_{i=1}^{k}\ln\;\;\ln\;Z_{i}+u$$

For the empirical estimation of the input–output relationship (as noted in Equation *7*) in milk production, we employed the following OLS regression model:

(8) $${\ln\;Y\;}_{i}=\beta_{1\;}\;\ln\;{fodder\;expenditure}_{i}+\beta_{2\;}\;\ln\;{concentrate\;\&\;feed\;expenditure}_{i}+\beta_{3}\;\ln\;t{ransportation\;cost\;of\;raw\;inputs}_{i}+\beta_{4}\;\ln\;{Other\;expenditure}_{i}+\underline{\delta }\underline{X}+\mu_{i}$$

In Equation *8*, *Y* represents the reported quantity of milk produced in the household (i represents the households and $\mu_{i}$ is the error term). In this framework expenditure on fodder, concentrate and feed, transportation cost of raw inputs and other expenditures incurred on cattle rearing (such as veterinary expenditures) were included as the primary inputs. The confounding effects of the number of cattle owned by the household, agriculture land ownership, and an indicator variable for cattle shed facility were considered in the model (represented by vector *X* in Equation *8*). Because the producing households did not report data on labor for animal husbandry activities, we did not consider labor cost as an input. Moreover, the survey data did not permit us to impute the labor cost of a family member in milk production, as the information regarding which member participated and the number of hours spent were not available from the survey data.

All analyses were performed using Stata version 17 [17], and the regression coefficients with 95% confidence interval (CI) are reported. Clustering was accounted for at village level in all the above analysis by considering cluster robust variance estimates.

## Results

**FIGURE 2:**
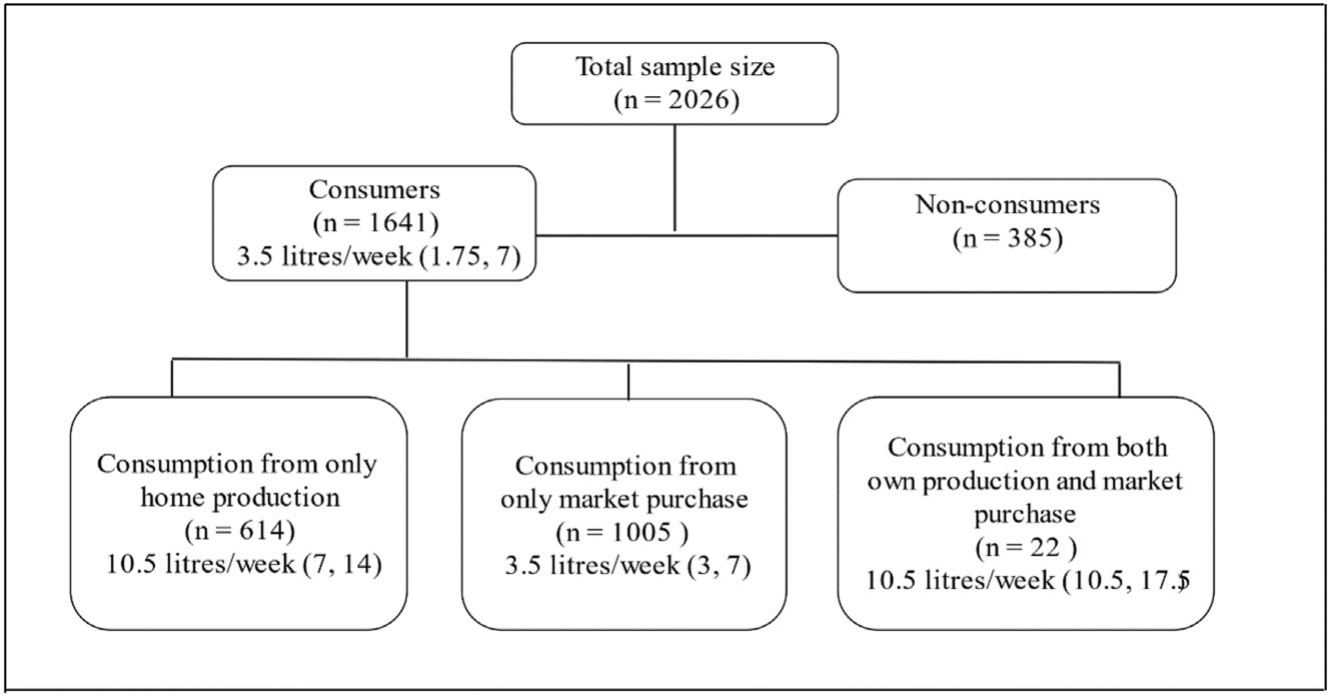
Classification of households based on the source of milk consumption. Median (quartile 1, quartile 3) intake of milk across various sources of consumption- home production, market purchase and both the sources. “n” represents the number of households in each category.

The characteristics of the 2026 households from the Round 1 consumption survey are presented in Table 1. The median size of the family and the number of children were 6 and 3, respectively. The median reported MPCE (which is proxy for income level of the household) is Indian rupee (INR) 1849 (IQR: 1321, 2636) (i.e., USD 22.63). Milk was consumed by 81% (1641 households) of the households, and the median (IQR) per capita milk consumption per day was 83 (IQR: 11.9, 166) mL. Figure 2 depicts the median (IQR) intake of milk classified by the source of intake, that is, own production and market purchases. Of 1641 milk-consuming households, 614 households consumed milk from their own production, 1005 households relied on market purchases for milk consumption, and 22 households consumed from both own production and market purchases. Households that consumed milk from their own production and from both sources (own production and market purchase) had higher per capita median household milk intake of 10.5 L/wk (1.75 L/wk), compared with 3.5 L/wk (0.58 L/wk) reported by those households that consumed from market purchase alone.

**TABLE 1:** Characteristics of households in Round 1 milk consumption survey

Characteristic	Summary
Number of households	2026
Household size	6 (5, 8)
Number of children in the household	3(2, 4)
Number of adults in the household	3 (2, 5)
Monthly per capita expenditure	1849 (1321, 2636)
Household owns land	95%
Household consumes milk	81%
Price of milk purchased (INR)	40 (40, 40)
Household consumption of milk from own production (L/wk)	14 (12, 28)
Household consumption of milk from market purchase (L/wk)	3.5 (3, 7)
Household consumption of milk from both production and market purchase (L/wk)	10.5 (10.5, 17.5)
Household consumption of other milk products (kg/wk)	0 (0, 0.5)
Household consumption of rice (kg/wk)	8.25 (7.5, 11.75)
Household consumption of wheat (kg/wk)	7.6 (6, 11.37)
Household consumption of vegetables (kg/wk)	1.25 (0.5, 2.5)
Household consumption of pulses (kg/wk)	1.5 (1,2.25)
Household consumption of eggs (numbers per week)	3 (2, 5)
Household consumption of fish (kg/wk)	0.37 (0.25, 0.5)
Household consumption of meat (kg/wk)	0.5 (0.25, 0.75)

All data represented as median (first quartile, third quartile) unless otherwise specified.

Abbreviation: INR, Indian rupee (1 INR = 0.013 USD).

### Factors associated with milk consumption—availability and accessibility

The consumption of milk was higher in households that consumed from their own production (β: 7.1 L/wk; 95% CI: 6.1, 8.1) than those dependent on market purchases (Table 2). Households that consumed milk from both sources also had higher consumption (β: 5.2 L/wk; 95% CI: 4.6, 12.2) relative to consumption from market purchase. The regression coefficient from the model (β: −3.1 L/wk; 95% CI: −3.7, −2.5) for nonconsumers indicates that the household consumption from market purchase was 3.1 L/wk. These results indicate that own production of milk facilitated better household consumption than depending on market purchase. Furthermore, household milk consumption increased monotonically with wealth quintiles. Household consumption was also positively associated with the number of children and adults in the household, weekly household consumption of pulses, and land ownership. However, factors such as amount spent on the purchase of milk, household being beneficiary of subsidized food distribution, sex of household head, and highest educational attainment of females in the household were not associated with milk consumption. About 80% of the households were nonvegetarian.

**TABLE 2:** Availability and accessibility factors associated with household liquid milk consumption (liters/week)

Dependent variable: household milk consumption (L/wk)	All households (*n* = 1941)	Milk purchasing households (*n* = 951)
	Regression coefficient (95% CI)	Regression coefficient (95% CI)
Household consumption (consumption from market purchase)	Reference	—
Household consumption from own production	7.16 (6.19, 8.13)	—
Household consumption from both own production and market purchase	5.26 (1.65, 8.88)	—
Non consumers of milk	−3.15 (−3.73, −2.58)	—
Distance to market (km)	—	−0.001(−0.01, 0.005)
Location of the market (inside the village or not)	—	−1.14 (−3.58, 1.25)
Quintiles of MPCE		
First quintile	Reference	Reference
Second quintile	0.75 (0.21, 1.29)	1.39 (0.93, 1.94)
Third quintile	1.71 (1.04, 2.38)	1.65 (1.07, 2.44)
Fourth quintile	2.91 (1.99, 3.84)	2.88 (2.04, 3.74)
Fifth quintile	5.53 (4.08, 7.04)	4.11 (2.92, 5.42)
No. of children in household	0.45 (0.12, 0.79)	0.28 (0.05, 0.57)
No. of adults in household	0.87 (0.34, 1.41)	0.64 (0.26, 0.81)
Household consumption of rice (kg/wk)	−0.13 (−0.26, −0.01)	−0.08 (−0.21, 0.04)
Household consumption of wheat (kg/wk)	0.01 (−0.12, 0.15)	−0.04 (−0.18, 0.1)
Household consumption of vegetables (kg/wk)	0.27 (−0.33, 0.88)	−0.25 (−0.55, 0.04)
Household consumption of pulses (kg/wk)	0.62 (0.003, 1.23)	0.12 (−0.19, 0.44)
Household weekly consumption of eggs	0.09 (−0.09, 0.27)	−0.03 (−0.12, 0.07)
Household consumption of fish (kg/wk)	−0.86 (−2.23, 0.51)	−0.71 (−1.67, 0.25)
Household consumption of meat (kg/wk)	−0.59 (−1.58, 0.41)	−0.02 (−0.63, 0.44)
PDS beneficiary	−0.57 (−1.21, 0.07)	−0.39 (−0.96, 0.13)
Household owns >0.06 acres (median land holding) of agricultural land	0.79 (0.21, 1.37)	0.67 (0.07, 1.19)
Education attainment of the household head (secondary and higher)	0.69 (0.03, 1.34)	0.84 (0.28, 1.75)
Female as household head	0.48 (−0.24, 1.18)	1.49 (−1.55, 4.54)

All households: Linear regression of milk consumption with all listed variables in 1941 households with complete data on listed variables. Milk-producing households: Linear regression of milk consumption with all listed variables in 961 households that purchased milk and had complete data on listed variables. Regression coefficients were adjusted for all the variables listed in the table and clustering accounted at village level.

Abbreviations: CI, confidence interval; INR, Indian rupee (1 INR = 0.013 USD); MPCE, monthly per capita expenditure; PDS, public distribution system providing rice and wheat in Bihar state.

The role of accessibility of milk for consumption was examined in a subsample of 951 households that purchased milk from the market (Table 2). Accessibility was examined based on 2 indicators: distance to market (source of purchase of milk) and location of the market (the binary variable indicating the source of purchase being inside or outside the village). The results indicated that neither distance to market nor location of the market was associated with milk consumption among the households that purchased milk.

### Association between milk consumption from own production and household diet diversity

We found that household consumption of milk from own production was not significantly associated with household diet diversity score (Supplemental Table 2). The households in higher income quintiles were more likely to have higher dietary diversity. Furthermore, PDS beneficiary households had higher dietary diversity.

### Characteristics of milk-producing households

The characteristics of milk-producing households are reported in Table 3 based on the Round 2 survey conducted in 350 milk-producing households. The majority of producing households (87%) owned 1 cow/buffalo, and 72% had a cattle shed facility. Only a very small share (1.7%) of milk producers were members of cooperative societies. The median quantity of milk produced was 15 L/wk (IQR: 8.7, 30). The median household consumption of milk increased with production tertiles (7, 14, and 21 L/wk in tertile 1, tertile 2, and tertile 3, respectively). Among the 350 producers, 68% were nonsellers, and the remaining 32% were sellers (112 households). The median reported sale price/liter of milk was 40 INR (∼0.5 USD), and the median distance to the source of sale was 0.6 km. The survey also provided information about the key challenges faced by households for not producing milk. Shortage of space and capital scarcity were the 2 critical reasons reported for not producing milk (Supplemental Table 3).

**TABLE 3:** Characteristics of milk-producing households based on Round 2 survey (*n* = 350)

Characteristics	Summary
Household characteristics	
Household owns only 1 cow	87.1%
Cattle shed facility	72.3%
Member of cooperative society	1.7%
Milk producers own land greater than the median (0.093 acres)	71.7%
Household production	
Quantity of milk produced in a week^1^ (L)	15 (8.75, 30)
Tertile 1	10 (7.5, 15)
Tertile 2	22.5 (22.5, 22.5)
Tertile 3	37.5 (30, 45)
Household consumption from milk produced	
Quantity of milk consumed by producers^1^ (L/wk) (*n* = 350 households)	10.5 (7, 15)
Quantity of milk consumed from own production Tertile 1^1^ (L/wk) (*n* = 185 households)	7 (3.5, 14)
Quantity of milk consumed from own production tertile 2^1^ (L/wk) (*n* = 63 households)	14 (7, 21)
Quantity of milk consumed from own production tertile 3^1^ (L/wk) (*n* = 84 households)	21 (12, 28)
Quantity of milk sold	
Number of households that sold milk	112
Quantity of milk sold in a week^1^ (L)	15 (7.5, 22.5)
Tertile 1	7.5 (7.5, 7.5)
Tertile 2	15 (15, 15)
Tertile 3	30 (22.5, 37.5)
Distance to the source of sale^1^ (km)	0.6 (0.5, 1.55)
Sale price^1^ (per L)	40 (40, 40)

1 Data presented as median (first quartile, third quartile).

Sellers were classified into small and large sellers based on the median quantity of milk sold. If milk sold was higher than the median quantity sold (15 L/mo), then they were classified as large sellers. Conversely, households that sold lower than the median were considered small sellers. There were 73 small sellers and 39 large sellers in our sample of households. The Indian Council of Medical Research National Institute of Nutrition [18] specifies 300 mL for adults and 500 mL for children (1–18 y) as a daily recommended minimum intake of milk. Aggregating these recommended values for a median-sized household (household size 6, with 3 adults and 3 children) gives the minimum recommended milk intake for the household per month as 16.8 L.

**FIGURE 3:**
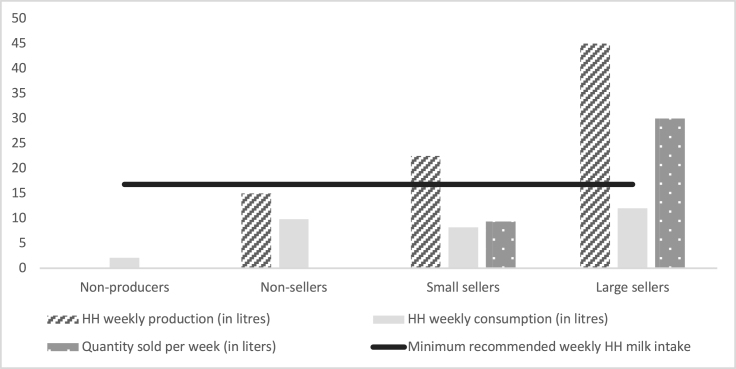
Milk consumption and production pattern across producers and nonproducers. Y-axis represents the quantity of milk in liters per week. The median quantity of milk produced, consumed, and sold in a week for nonproducers, nonsellers, small sellers, and large sellers are depicted in the figure. The Indian Council of Medical Research Dietary Guidelines for Indians (ICMR National Institute of Nutrition, 2020) recommends the minimum milk intake for adults is 300 mL/d and for children 500 mL/d. Aggregating the recommended values for a median-sized household (household size 6 with 3 adults and 3 children), gives the minimum recommended milk intake for the household per week as 16.8 L (represented by the horizontal line in the figure). HH, household.

Across all categories, household consumption of milk was lower than the minimum recommended intake (Figure 3). Both small and large sellers had higher production of milk than the minimum recommended levels of consumption. However, there was a preference for the sale of milk over consumption among the sellers. For instance, among the larger sellers, the sale quantity (30 L/wk) was 2.5 times the household consumption level (12 L/wk). Small sellers also had a similar preference toward sale over milk consumption (median quantity sold was around 9.3 L of 22.5 L of milk production).

### Association between milk consumption and quantity produced

In the subsample of milk producers (*n* = 350), household milk consumption was positively associated with milk production (Table 4). Households in higher production tertiles had higher consumption than households in production tertile 1. For instance, households in tertile 3 (with median production of 37.5 L/wk, IQR: 30, 45) had higher consumption (β: 14.3; 95% CI: 12.1, 17.2) than the lowest tertile (with median production of 10 L/wk, IQR: 7.5, 15). However, milk consumption was lower in households that sold milk (β: −6.8; 95% CI: −9.1, −4.5), and the consumption decreased with increased quantity of sale. The quantity of consumption was lower by 4.8 and 8.8 L/wk in the second and third tertiles than that of the first tertile of milk sold (Table 4), and the quantity of consumption was 3.2 L/wk higher in the households that did not sell milk than that of the first tertile of milk sold. Milk consumption significantly increased with the number of children and number of adults in the household. The consumption of milk was also higher in the highest income quintile.

**TABLE 4:** Factors associated with household milk consumption (liters/month) in milk-producing households

Dependent variable: Household milk consumption (L/wk)	Association with production quantity (*n* = 350) Regression coefficient (95% CI)	Association with sale quantity (*n* = 350) Regression coefficient (95% CI)
Tertiles of milk production		
Tertile 1: median production = 10 L/wk	Reference	Reference
Tertile 2: median production = 22.5 L/wk)	7.13 (4.88, 9.37)	7.49 (5.27, 9.71)
Tertile 3: median production = 37.5 L/wk	14.37 (12.1, 17.28)	16.85 (13.89, 19.81)
Household sold milk (yes)	−6.83 (−9.1, −4.57)	—
Tertiles of quantity of milk sold		
Tertile 1: median quantity = 7.5 L/wk	—	Reference
Tertile 2: median quantity = 15 L/wk	—	−4.82 (−9.22, −0.42)
Tertile 3: median quantity = 30 L/wk	—	−8.87 (−12.74, −5.01
Nonsellers	—	3.2 (1.1, 5.3)
Quintiles of MPCE		
First	Reference	Reference
Second	2.46 (−0.21, 5.14)	2.3 (−0.28, 4.88)
Third	0.72 (−1.51, 2.95)	0.93 (−1.19, 3.06)
Fourth	1.92 (−0.21, 4.05)	1.75 (−0.41, 3.92)
Fifth	4.68 (1.75, 7.61)	4.19 (1.34,7.05)
Number of children	0.63 (−0.01, 1.28)	0.65 (0.01,1.3)
Number of adults	0.55 (0.17, 0.93)	0.47 (0.08,0. 85)
Household-produced cereals (yes)	1.32 (−1.06, 3.71)	0.61 (−1.6, 2.81)
Household-produced pulses (yes)	−1.84 (−3.71, 0.02)	−1.94 (−3.71, 0.17)
PDS beneficiary	−1.72 (−3.67, 0.21)	−1.37 (−3.23, 0.47)
Household owns >0.06 acres (median land holding) of agricultural land	0.06 (−2.07, 2.18)	1.01 (−1.04, 3.06)
Education of household head (secondary and higher)	−0.02 (−1.89, 1.8)	0.08 (−1.72, 1.9)
Female as household head	−0.02 (−2.2, 2.16)	0.15 (−1.82, 2.12)

Regression coefficients were adjusted for all the variables listed in the table. Number of observations (milk-producing households) is 350.

Abbreviations: CI, confidence interval; MPCE, monthly per capita expenditure, PDS, public distribution system.

### Factors associated with milk production

The analysis of the log of quantity of milk produced against the monthly cost for fodder, concentrate and feeds, transportation cost of raw inputs, veterinary expenses, number of cattle, and indicator variables for both cattle shed and land ownership (Table 5). Among these, expenditure on concentrate/feed and the number of cattle were positively associated with quantity of milk production. None of the other factors was significantly associated with raising the quantity of milk production in producing households.

**TABLE 5:** Input factors associated with quantity of milk production in producing households (*n* = 350)

Dependent variable: Log of the quantity of milk produced	Coefficient (95% CI)
Fodder (green and dry) expenditure/month^1^	−0.01 (−0.03, 0.01)
Concentrate and feed expenditure/month^1^	0.03 (0.01, 0.06)
Transportation cost of raw inputs per month^1^	−0.01 (−0.05, 0.04)
Veterinary expenditure per month^1^	0.03 (−0.01, 0.07)
Cattle shed facility provided (0 – no, 1 – yes)	0.07 (−0.11, 0.26)
Number of cattle owned by household	0.37 (0.14, 0.61)
Cultivable land owned higher than the median (0.094 acres)	0.17 (−0.03, 0.37)

Abbreviation: CI, confidence interval.

1 Log to the base 10 of these variables is considered in the model.

## Discussion

A household’s decision to consume their food from own production or to purchase from the market has important implications for nutrition [3,19]. In recent years, there has been a growing interest in exploring the food consumption–production linkages among farming households [20,21]. However, these studies largely focus on the consumption pattern of foods of plant origin. The relationship between consumption–production linkages in animal-sourced foods such as milk is inadequately explored in the literature [22]. The present study examines the consumption–production pattern of milk among rural households of Bihar state in India. The quantity of milk consumed in milk-producing households was higher, and within producing households, it was positively associated with the quantity produced and negatively associated the quantity of milk sold by the household. The quantity of milk consumed per capita on average was median 83 (IQR: 11.9, 166) mL/d in the second round (83.3; IQR: 41.6, 166.6 mL in the first round) and was slightly higher in the milk-producing households, at median 250 (IQR: 166, 357) mL/d, but was considerably lower than the recommended quantity of milk to be consumed (for adults 300 mL/d and for children 500 mL/d) as per the Indian dietary recommendations. This was due to a preference for the sale of milk over consumption in producing households.

The National Institute of Nutrition recommends that an Indian household of 6 members (4 adults and 2 children) should consume 12.6 L of milk per week to meet the nutrient requirements in a balanced manner. However, the median consumption of milk was only 3.5 L/wk [18]. The consumption was higher by ∼7 L/wk in households that consumed milk from own production than those who relied on markets. Our results are in line with other studies that find household production is a key driver of milk consumption. In a study [6] that explored patterns of food consumption in 12 selected villages of eastern India, the authors found that the Bihar villages were self-reliant for milk, with 42% to 86% of consumption arising from home production, explaining their higher milk consumption relative to the rest of the villages.

The results also showed that milk consumption increased with the quantity of milk produced. Similarly, studies in Uganda [23] and Tanzania [24] found evidence on dairy production and increased milk consumption. The consumption level of nonsellers was high even though they were in the lower quintiles of production. In contrast, milk sellers tended to have lower milk consumption despite having higher production than nonsellers. Quantity of milk consumption reduced with increase in quantity of milk sold by the households. In a similar study based on the state of Uttaranchal, India, Bohra et al. [25] note that milk as a source of income often prompted production for sale and led to a reduction in the milk retained for consumption in the household. Njarui et al. [26] also reported in a study in Kenya that as production increased in households, consumption decreased, implying that dairy farming was for market sale rather than for household consumption. Alternatively, in this cross-sectional data analysis, households could have produced more milk because they consumed more, as unidirectional causation cannot be established.

Examining a large number of studies on the linkages between agriculture and nutrition, Ruel et al. [3] observed that cattle ownership is consistently associated with greater milk intake and dietary diversity. Our results imply that one of the major factors that was associated with production of milk was the number of cows owned by the household. Milk production in Bihar is dominated by smallholder dairy farms, with small average herd size of 1.5 cows/buffaloes per household [27]. Therefore, the production is limited and insufficient to generate income and simultaneously meet the nutritional requirements of the household.

In a study of smallholder dairy farms in Bihar [27], dairy cooperative membership raised milk production yield (net returns per liter) and enhanced smallholder dairy farmers’ income. Our study revealed that only 1.7% of producers are members of cooperative societies. Thus, in Bihar, there is a need to focus on dairy cooperatives expansion to raise milk production and increase returns to smallholder dairy farmers.

Previous studies on nutrition suggest that better market accessibility positively affects diet diversity [28,29]. In this study, 96% of the households purchased milk either from neighboring households or from door-to-door sellers, and therefore, even for purchasing households, milk was accessible. Therefore, accessibility (captured by distance to market and location of the market) was not significantly associated with milk consumption. However, affordability was significantly associated with household milk consumption. In Kenya, richer households consume significantly more milk than poor households [26]. In a related study in India, Kadiyala et al. [30] found that households in higher income groups had higher budgetary shares of milk. Apart from income, other socioeconomic and demographic factors, such as the number of children and adults in the household and the size of the land, play a significant role in milk consumption. This population considered milk as a good source of nutritious food for children [31]. Therefore, the consumption of milk increased with the number of children in the household. This was confirmed with an analysis of per capita milk consumption by the household as the dependent variable. Number of children in the household was positively associated with per capita consumption as well, thus confirming that number of children is not a proxy for household size and the association observed is not spurious. Through meta-analysis of factors influencing milk consumption, Kapaj and Deci [32] found that studies from different regions of the world report that size of young children, property, and income are common factors significantly associated with quantity of milk consumption in the household.

Literature suggests that diversified farm production contributes to the dietary diversity of rural households [9,33]. Household production ensures availability and provides additional income from sales revenue that can be used to purchase essential foods from the market [16]. We did not examine production diversity in this study. Nevertheless, milk consumption from own production was not associated with household dietary diversity.

There are some limitations to the study. A clearer understanding of barriers to own production may have explained why more households were not producing milk, although there was a preference for home-produced milk. The cost of production was not complete and therefore limited in utility. The association between home production and consumption also depends on climate risk as it can affect diary cow milk production productivity [34], which was not accounted for in this study due to limited data availability. We also did not have data on storage and refrigeration facility for milk in the household, which may have influenced the association of milk consumption and production.

The present study contributes to the scarce empirical evidence related to the production–consumption linkage of milk in India. The study provides evidence that household production of milk can significantly increase milk consumption even when the production is small scale. However, household sale of milk can impinge on its consumption. Even for nonproducing households, market accessibility is not a binding constraint for milk consumption. Finally, households that produced milk did not have better diet diversity.

The share of milk in agricultural production had been increasing relative to that of cereals in India, consistent across South Asia. For instance, the milk production in India and Pakistan has grown by 3% or more, whereas cereals output growth has been slower [35]. Policies advocating cattle farming among agricultural households have the potential to improve quantity of milk production and thereby consumption among small farm households. Subsidized feed supplies for poor households could be an effective policy strategy for improving milk production. Public support and institutional innovations seem to be associated with expanded provisions of agricultural finance and insurance in milk production. Marketing institutions including contract farming and cooperatives have become increasingly important for production and marketing of milk [27,35]. Our survey reports that capital scarcity was one of the most pressing concerns for farmers to start milk production. Availing more loans to support animal husbandry can raise milk production as well as improve milk consumption.

## Author contributions

The authors’ responsibilities were as follows – RR and TT wrote the initial draft. SS, AVK and TT conceptualized the study. RR,SS and TT performed the statistical analysis. AVK and PW reviewed and revised the manuscript. All authors read and approved the final manuscript.

### Conflict of interest

The authors report no conflicts of interest.

### Funding

This work was supported by the Bill & Melinda Gates Foundation, Seattle, WA [Grant Number: OPP1194964]. The Bill & Melinda Gates Foundation did not have any role in the study design; collection, analysis, and interpretation of data; writing of the report; or the decision to submit the article for publication.

### Data availability

The data that support the findings of this study are available from the corresponding author upon reasonable request.
