# Supplementary material for: Evidence on Milk Consumption and Production Linkages from Rural Bihar, India
Source: Curr Dev Nutr. 2024 Feb 24;8(4):102122. doi: 10.1016/j.cdnut.2024.102122 (PMC11043808; doi:10.1016/j.cdnut.2024.102122)
Supplement: Multimedia component 1 [file mmc1.docx]

**Supplemental Table 1** Characteristics of milk producers in Round 1

| **Characteristic of milk producers** | **Round 1 -summary of all milk producers** | **Round 1 -Summary of milk producers sampled in Round 2** |
| --- | --- | --- |
| Number of households | 614 | 350 |
| Household size | 7(5, 9) | 7(5, 9) |
| Number of children in the household | 3(2,4) | 3(2, 4) |
| Number of adults in the household | 4(3, 5) | 4(3, 6) |
| Monthly per capita expenditure, MPCE | 2105(1430, 3007) | 2136(1533, 3047) |
| Household owns land (%) * | 77 | 71 |
| Household consumption of milk (litres /week) | 7 (3.5, 14) | 10.5(7, 14 ) |

All data represented as median (1st quartile, 3rd quartile). * implies data reported as percentage and household denotes households.

**Supplemental Table 2.** Association between milk consumption from own production and diet diversity

| **Dependent variable: Diet Diversity (1-if HDDS greater than 10, 0-otherwise)** | **Round 1**  **(n=1941)** |
| --- | --- |
|  | **Regression Coefficient**  **[95% Conf. Interval]** |
| Household milk consumption from own production  (yes) | 0.02 (-0.11, 0.15) |
| Quintiles of MPCE (First quintile) | Reference |
| Second quintile | 0.22 ***(0.05,0.39) |
| Third quintile | 0.49*** (0.32,0.67) |
| Fourth quintile | 0.51*** (0.31,0.7) |
| Fifth quintile | 0.66*** (0.47,0.86) |
| No. of children in household | 0.03 (-0.002,0.07) |
| No. of adults in household | 0.02 (-0.01,0.06) |
| PDS beneficiary | 0.18** (0.06,0.31) |
| Household owns more than 0.06 acres (median land holding) of agricultural land | -0.04 (-0.16,0.07) |
| Education attainment of the household head (secondary and higher) | 0.03 (-0.1,0.16) |
| Woman as household head | -0.05 (-0.22,0.12) |

Note: MPCE- Monthly per capita expenditure. PDS-Public Distribution System providing rice and wheat in Bihar state. *** and ** represent p < 0.01 and p<0.05, respectively.

**Supplemental Table 3**. Reasons reported by non-producing households for not engaging in milk production

| **Reasons** | **Percentage of households reporting the reason (n=518)** |
| --- | --- |
| Production inputs challenge | 18.53 |
| Shortage of capital | 65.64 |
| Shortage of time due to current occupation | 13.5 |
| Shortage of space | 78.19 |
| No knowledge /training for production | 8.4 |

Note: Households had reported one or more reasons for not-producing milk, hence the aggregation of percentage
